# Progress Toward Poliomyelitis Eradication — Nigeria, January–December 2017

**DOI:** 10.15585/mmwr.mm6708a5

**Published:** 2018-03-02

**Authors:** Omotayo Bolu, Chimeremma Nnadi, Eunice Damisa, Fiona Braka, Anisur Siddique, W. Roodly Archer, Philip Bammeke, Richard Banda, Jeffrey Higgins, Aboyowa Edukugo, Gatei wa Nganda, Joseph C. Forbi, Hongmei Liu, Saheed Gidado, Mohammed Soghaier, Richard Franka, Ndadilnasiya Waziri, Cara C. Burns, John Vertefeuille, Eric Wiesen, Usman Adamu

**Affiliations:** ^1^CDC Nigeria Country Office, Abuja, Nigeria; ^2^Global Immunization Division, CDC; ^3^Polio Emergency Operations Center, National Primary Health Care Development Agency, Abuja, Nigeria; ^4^Expanded Program on Immunization, World Health Organization, Nigeria Country Office, Abuja, Nigeria; ^5^United Nations Children’s Fund, Nigeria Country Office, Abuja, Nigeria; ^6^National Stop Transmission of Polio Program, Africa Field Epidemiology Network, Nigeria Office, Abuja, Nigeria; ^7^Division of Viral Diseases, National Center for Immunization and Respiratory Diseases, CDC.

Nearly three decades after the World Health Assembly launched the Global Polio Eradication Initiative in 1988, four of the six World Health Organization (WHO) regions have been certified polio-free (*1*). Nigeria is one of three countries, including Pakistan and Afghanistan, where wild poliovirus (WPV) transmission has never been interrupted. In September 2015, after >1 year without any reported WPV cases, Nigeria was removed from WHO’s list of countries with endemic WPV transmission (*2*); however, during August and September 2016, four type 1 WPV (WPV1) cases were reported from Borno State, a state in northeastern Nigeria experiencing a violent insurgency (*3*). The Nigerian government, in collaboration with partners, launched a large-scale coordinated response to the outbreak (*3*). This report describes progress in polio eradication activities in Nigeria during January–December 2017 and updates previous reports (*3*–*5*). No WPV cases have been reported in Nigeria since September 2016; the latest case had onset of paralysis on August 21, 2016 (*3*). However, polio surveillance has not been feasible in insurgent-controlled areas of Borno State. Implementation of new strategies has helped mitigate the challenges of reaching and vaccinating children living in security-compromised areas, and other strategies are planned. Despite these initiatives, however, approximately 130,000–210,000 (28%–45%) of the estimated 469,000 eligible children living in inaccessible areas in 2016 have not been vaccinated. Sustained efforts to optimize surveillance and improve immunization coverage, especially among children in inaccessible areas, are needed.

## Security Situation

**Figure 1:**
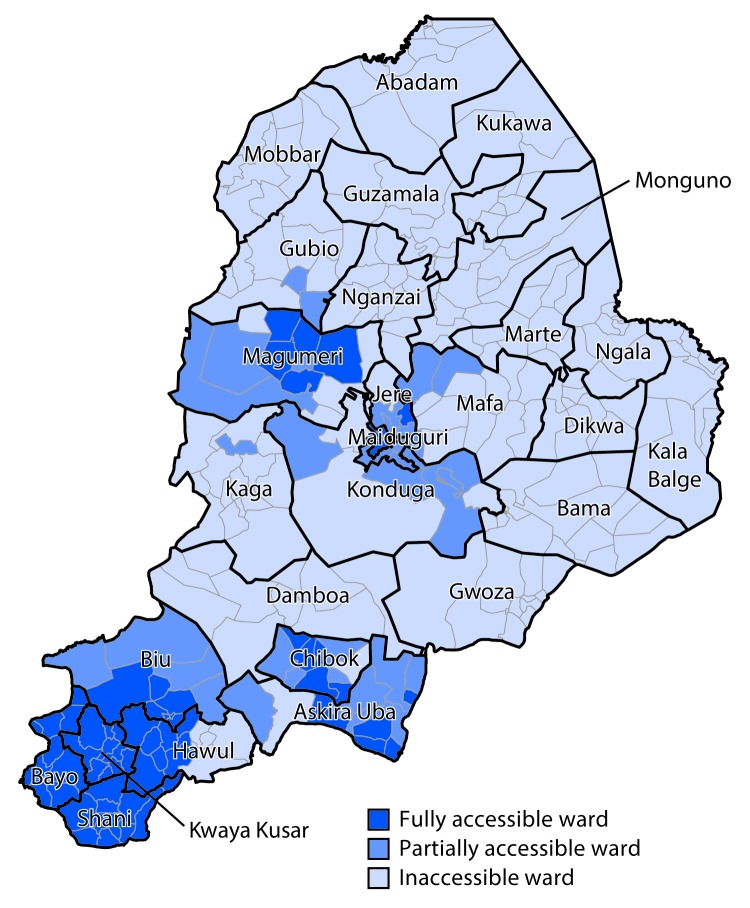
Accessibility of local government areas to polio eradication program personnel, by ward — Borno State, Nigeria, September 2015

During the past 8 years, Borno State in northeastern Nigeria has been at the center of an insurgency that has affected other Nigerian states including Adamawa, Gombe, and Yobe and the neighboring Lake Chad Basin countries of Cameroon, Chad, and Niger. Insecurity in this region has led to a major humanitarian emergency, with forced displacement of an estimated 2.1 million persons within Nigeria and 200,000 refugees seeking shelter in other countries (*6*). At the height of the insurgency in 2015, 60% of settlements were inaccessible for implementation of vaccination and surveillance activities (Figure 1). Security assessments conducted during December 2017 in Borno State indicate that of the 27 districts (local government areas [LGAs]), eight (30%) are fully accessible to polio eradication program personnel, 17 (63%) are accessible only by polio eradication program personnel with military escorts, and two (7%) are accessible only by combat-ready military personnel. Overall, an estimated 30% of subdistrict level communities (settlements) in Borno State were fully inaccessible to personnel in the Nigeria polio eradication program. Analyses of satellite imagery conducted in October 2017 estimated that approximately 130,000–210,000 (28%–45%) of the estimated 469,000 children aged ≤5 years living in inaccessible areas in Borno State in 2016 had not been reached by polio vaccination or surveillance efforts.

## Poliovirus Surveillance

**Acute Flaccid Paralysis Surveillance.** During 2016 and 2017, Nigeria met major acute flaccid paralysis (AFP) performance indicators for all states, including the nonpolio AFP (NPAFP) rate (which assesses surveillance system sensitivity) and stool adequacy (which assesses the timeliness and appropriateness of investigation of suspected cases) (*7*). In Borno State, NPAFP rates of 27.0 (2016) and 24.5 (2017) cases per 100,000 children aged <15 years were reported, exceeding the target of three per 100,000. Among all persons with reported AFP, 95% in 2016 and 90% in 2017 had two adequate stool specimens collected 24–48 hours apart and ≤14 days after paralysis onset (target = 80%). However, the population in the security-compromised areas have not been accessible for surveillance efforts. In addition, concerns about the quality of case detection and investigation in accessible areas of Borno State and Adamawa, Taraba, and Yobe states in the Northeast and Kaduna and Sokoto states in the Northwest were identified through surveillance assessments in 2017, and indicate the potential to miss detection of poliovirus transmission elsewhere in Nigeria.

Surveillance system strengthening measures were implemented in 2016 and 2017, including increased AFP case searches among camps for internally displaced persons in Borno State, engagement of community informants from inaccessible areas, and retrospective active case searches in newly accessible areas. To improve timeliness and accuracy of AFP case reporting in geographic hard-to-reach areas, a mobile phone application, Auto-Visual AFP Detection and Reporting (AVADAR), was implemented, starting in selected high priority LGAs in Borno State and Adamawa, Sokoto, and Yobe states (*8*). Despite these efforts, 25 of 27 LGAs in Borno State had settlements that were inaccessible for surveillance in 2016 and 2017, including all of Abadam and Marte LGAs (Figure 1).

**Environmental Surveillance.** Environmental surveillance, through testing wastewater sampled at selected sites, can be a sensitive supplement to AFP surveillance for detection of polioviruses. During 2017, the number of such sites increased 33%, from 56 in 30 LGAs to 70 in 35 LGAs. As of December 2017, 18 of the 37 state-level jurisdictions in Nigeria had at least one environmental surveillance site. In addition, the frequency of sample collection at most surveillance sites has increased from once to twice monthly in many states, and more frequently in Borno State and some other high-risk areas. Seven wastewater collection sites can be found in the metropolitan area of Maiduguri, the capital of Borno State. In 2017, no circulating vaccine derived poliovirus type 2 (cVDPV2) or WPV isolates were detected through environmental surveillance.

## WPV and cVDPV Cases

After the switch from trivalent oral polio vaccine, which contains polio vaccine virus types 1, 2, and 3 to bivalent oral polio vaccine (bOPV) (types 1 and 3) in April 2016, enhanced laboratory testing using viral sequencing methods for type 2 poliovirus isolates was introduced to improve laboratory case detection. Since September 2016, no WPV cases have been reported in Nigeria. A cVDPV2 case was last identified in Sokoto in October 2016.

## Vaccination Activities

During 2017, eight mass vaccination campaigns (supplementary immunization activities [SIAs]) were conducted in Nigeria (Table). The first SIA in January was part of a response to the detection of cVDPV2 isolates in Borno State in March and August 2016 and in Sokoto in October 2016 (*3*). Two national-level SIAs were conducted in March and April; approximately 58 million children were vaccinated during each round. SIA performance was evaluated using lot quality assurance sampling (LQAS) methodology, which provides a quick and reliable immunization campaign assessment; >90% of LGAs surveyed passed the 80% LQAS threshold.

**TABLE:** Polio supplementary immunization activity dates, antigen types, coverage, and reported lot quality assurance sampling results — Nigeria, 2017

SIA date in 2017	Vaccine antigen type	Target area	No. children vaccinated	LGAs achieving ≥90% coverage on LQAS* (%)
Jan 28–31	mOPV2	18 northern states^†^	32,360,489	89
Feb 25–28	bOPV	14 states at the highest risk for polio^§^	25,350,055	87
Mar 25–28	bOPV	All 36 states + FCT	57,937,250	75
Apr 29–Aug 22^¶^	bOPV	All 36 states + FCT	57,928,320	77
May 20–30**	IPV + mOPV2	Sokoto	463,963 (IPV) 1,893,914 (mOPV2)	91
Jul 8–11	bOPV	18 northern states^†^	32,449,576	85
Oct 6–24^††^	bOPV	18 northern states^†^	31,242,217	78
Nov 4–14	bOPV	7 highest priority states^§§^	9,847,162	89

**Abbreviations:** bOPV = bivalent oral poliovirus types 1 and 3; FCT = federal capital territory; IPV = inactivated polio vaccine; LGAs = local government areas; LQAS = lot quality assurance sampling; mOPV2 = monovalent oral poliovirus type 2; SIA = supplemental immunization activity.

* ≥90% coverage achievement pass mark on LQAS set by Nigeria polio program.

^†^ 18 states included Abuja and the Federal Capital Territory, Adamawa, Bauchi, Borno State, Gombe, Jigawa, Kaduna, Kano, Katsina, Kebbi, Kogi, Nasarawa, Niger, Plateau, Sokoto, Taraba, Yobe, and Zamfara.

^§^ 14 states included Abuja and the Federal Capital Territory, Adamawa, Bauchi, Borno State, Gombe, Jigawa, Kaduna, Kano, Katsina, Nasarawa, Sokoto, Taraba, Yobe, and Zamfara.

^¶^ Campaign staggered across states to improve effectiveness and quality.

** Response to vaccine-derived poliovirus isolation.

^††^ Campaign in Borno State coordinated with other Lake Chad Basin countries.

^§§^ Seven states included Adamawa, Bauchi, Borno, Gombe, Sokoto, Taraba, and Yobe.

In collaboration with the Nigerian military, two measures have been employed to increase poliovirus immunity among children in insecure areas of Borno and Yobe states. The Reaching Every Settlement initiative engages civilian vigilante and military support to reach children in settlements in which vaccinators require security escorts. During 2017, approximately 251,000 children in 2,921 settlements were vaccinated during 16 Reaching Every Settlement rounds. The Reaching Inaccessible Children initiative deploys military personnel with basic vaccination training to vaccinate children living in settlements that can only be accessed by combat-ready military personnel. During 2017, six Reaching Inaccessible Children rounds vaccinated 50,196 children in 1,412 inhabited settlements.

**Figure 2:**
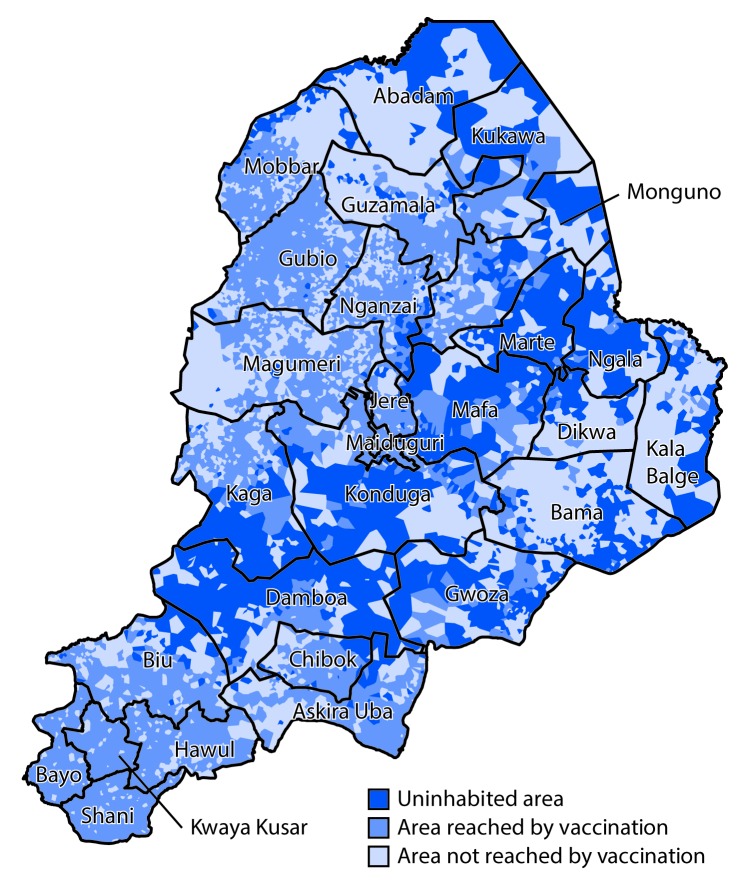
Polio vaccination coverage, by area — Borno State, Nigeria, August 2016–October 2017

Assessment of vaccination status of children arriving in camps for internally displaced persons from insurgent-held areas is conducted to help monitor progress in vaccinating children living in inaccessible areas. Other efforts aimed at improving population immunity include vaccination at markets and international borders and outreach to nomadic and migrant populations. In-depth analysis of satellite imagery to identify inaccessible areas (*9*) has facilitated identification and characterization of settlements and populations in areas inaccessible to polio program personnel (Figure 2), helping guide the implementation of targeted approaches to reach and immunize eligible children in these areas.

The routine immunization schedule for Nigeria includes bOPV at birth, followed by 3 additional bOPV doses at ages 6, 10, and 14 weeks. In 2015, a single dose of inactivated poliovirus vaccine was also included in the routine immunization schedule at age 14 weeks. During 2016–2017, a national coverage survey estimated that overall, 33% of children aged 12–23 months were fully vaccinated against polio, although large variations were observed by state, ranging from 7% in Sokoto and Yobe to 75% in Lagos.

## Discussion

Since the identification of four WPV cases in Borno State in 2016, the Nigerian polio program has intensified polio eradication activities, especially in areas experiencing insurgency. However, the ability of program personnel to implement eradication activities, including high quality surveillance and vaccination, has been limited because of ongoing insurgency-related inaccessibility to areas in Borno State and other states in the country’s northeast and northwest areas.

Because of military interventions against the insurgency, the percentage of settlements that are inaccessible to polio eradication personnel has been reduced from 60% in September 2015 to approximately 30% in December 2017. Implementation of the Reaching Every Settlement and Reaching Inaccessible Children strategies has helped reach some of the children living in areas inaccessible to house-to-house vaccination teams, and at least five contacts with children eligible for immunization in these areas are planned. Children reached by these initiatives need to be tracked to ensure receipt of the multiple OPV doses needed to complete the immunization series. Increased involvement of the military in implementing Reaching Every Settlement and Reaching Inaccessible Children strategies is planned in 2018 to reach as many children as possible in inaccessible areas.

The low OPV3 coverage among children aged 12–23 months in the 2016–2017 national vaccination survey reflects persistently poor delivery of routine immunization services, particularly in states in northeast and northwest Nigeria (*10*). A National Emergency Routine Immunization Coordinating Center has been commissioned to identify and implement strategies to increase vaccination coverage, starting in the poorest performing states. Understanding reasons for the persistently low coverage in the northeast and northwest areas despite years of investment is a major focus in planning remedial activities.

Surveillance activities have been strengthened since the last reported WPV case in Nigeria in September 2016, including expanding the number of environmental surveillance sites and increasing the number of surveillance community informants who reside in areas with limited access for polio program personnel in the states of Borno and Yobe to alert the program of potential AFP cases. Although national surveillance performance indicators are high, there are concerns about ongoing undetected poliovirus circulation in inaccessible areas; ongoing undetected poliovirus circulation is also possible in some accessible areas of Borno and other states where concerns about case investigation quality were identified.

Efforts to address the impediments created by insecurity and geographic access limitations continue to be implemented and expanded. Searching for recent AFP cases in security-compromised areas is one objective of the Reaching Every Settlement and Reaching Inaccessible Children initiatives. A commitment to strengthening routine and supplementary immunization coverage in all areas of the country is needed, as are efforts to ensure high quality surveillance.

**Summary**

**What is already known about this topic?**

In August 2015, the World Health Organization removed Nigeria from the list of polio-endemic countries because of the high likelihood that endemic wild poliovirus (WPV) circulation had been interrupted in Nigeria. However, during August and September 2016, four WPV cases were reported in Borno State, a northeastern Nigerian state experiencing protracted insurgency.

**What is added by this report?**

No WPV cases have been reported since September 2016. New strategies implemented by the Nigeria polio program have helped improve polio eradication activities, including those in areas with security challenges. However, approximately 28%–45% of eligible children living in the inaccessible areas have not been vaccinated, and surveillance has not been feasible in insurgent-controlled areas of Borno State.

**What are the implications for public health practice?**

Although access to communities for polio eradication activities continues to improve, approximately 30% of subdistrict level communities in Borno State remain inaccessible because of insurgency-related insecurity. Sustained efforts are needed to optimize surveillance and improve immunization coverage, especially among children in inaccessible areas.
